# Biomarkers for Immunotherapy Efficacy in Advanced Hepatocellular Carcinoma: A Comprehensive Review

**DOI:** 10.3390/diagnostics14182054

**Published:** 2024-09-16

**Authors:** Erfan Taherifard, Krystal Tran, Ali Saeed, Jehad Amer Yasin, Anwaar Saeed

**Affiliations:** 1 Department of Medicine, Division of Hematology & Oncology, University of Pittsburgh Medical Center, Pittsburgh, PA 15232, USA; erfantaherifard@gmail.com (E.T.); kbt19@pitt.edu (K.T.); jehadamerjehadyasin@gmail.com (J.A.Y.); 2 Department of Medicine, Ochsner Lafayette General Medical Center, Lafayette, LA 70503, USA; asaeedmd@gmail.com; 3 UPMC Hillman Cancer Center, Pittsburgh, PA 15232, USA

**Keywords:** hepatocellular carcinoma, biomarkers, immunotherapy, immune checkpoint inhibitors, programmed cell death 1, programmed cell death ligand 1, alpha-fetoproteins, cytokines, circulating tumor DNA, circulating neoplastic cells, microsatellite instability, tumor-infiltrating lymphocytes

## Abstract

Hepatocellular carcinoma (HCC), the most common primary liver malignancy and the sixth most common cancer globally, remains fatal for many patients with inappropriate responses to treatment. Recent advancements in immunotherapy have transformed the treatment landscape for advanced HCC. However, variability in patient responses to immunotherapy highlights the need for biomarkers that can predict treatment outcomes. This manuscript comprehensively reviews the evolving role of biomarkers in immunotherapy efficacy, spanning from blood-derived indicators—alpha-fetoprotein, inflammatory markers, cytokines, circulating tumor cells, and their DNA—to tissue-derived indicators—programmed cell death ligand 1 expression, tumor mutational burden, microsatellite instability, and tumor-infiltrating lymphocytes. The current body of evidence suggests that these biomarkers hold promise for improving patient selection and predicting immunotherapy outcomes. Each biomarker offers unique insights into disease biology and the immune landscape of HCC, potentially enhancing the precision of treatment strategies. However, challenges such as methodological variability, high costs, inconsistent findings, and the need for large-scale validation in well-powered two-arm trial studies persist, making them currently unsuitable for integration into standard care. Addressing these challenges through standardized techniques and implementation of further studies will be critical for the future incorporation of these biomarkers into clinical practice for advanced HCC.

## 1. Introduction

Primary liver cancers, particularly hepatocellular carcinoma (HCC), represent a major health burden globally in both terms of occurrence and cancer-related mortality. These cancers are currently ranked as the sixth most frequently diagnosed malignancy worldwide and constitute the third leading cause of cancer-related deaths, accounting for age-standardized rates of 9.5% and 8.7% of all cases, respectively [1,2]. Additionally, the incidence of liver cancer is expected to surge by 55.0% from 2020 to 2040, with an estimated 1.4 million new cases annually by 2040 [2]. Likewise, deaths from liver cancer are projected to rise by 56.4%, potentially resulting in 1.3 million deaths annually by 2040. This escalating burden, with HCC comprising about 75–85% of all primary liver cancer cases, driven by risk factors such as chronic hepatitis B and C, alcohol-related liver disease, and metabolic dysfunction–associated steatotic liver disease (MASLD), underscores the urgent need for enhanced prevention, early detection, and treatment strategies to mitigate its growing impact [3,4,5].

The current treatment landscape for HCC encompasses a broad spectrum of options, ranging from surgical resection and liver transplantation to locoregional therapies and systemic treatments [6]. The choice of treatment is carefully determined based on multiple factors such as the characteristics of the tumor and the liver’s functional reserve within a multidisciplinary care framework to tailor the most effective treatment plan for each patient [7,8]. For patients with advanced HCC, surgical interventions and locoregional therapies are generally not viable options due to the extensive spread of the disease and poor liver function. In these patients, systemic therapies may be employed to manage the disease. Currently, the approved systemic therapies for patients with advanced HCC fall into two main categories: immune checkpoint inhibitors (ICIs) and tyrosine kinase inhibitors (TKIs) [9,10]. ICIs work by targeting checkpoint proteins that typically inhibit the immune response, leading to an immunosuppressive tumor microenvironment in HCC and thereby, tumor immune evasion [11]. By blocking these checkpoints, such as programmed cell death protein 1 (PD-1) and its ligand (PD-L1), or cytotoxic T-lymphocyte-associated protein 4 (CTLA-4), ICIs re-engage the immune system to attack cancer cells. TKIs, on the other hand, target specific tyrosine kinases involved in various signaling pathways that play crucial roles in tumor growth and progression [12].

For nearly a decade, sorafenib, a TKI, was the only systemic therapy available for patients with advanced HCC. However, significant advancements have since transformed systemic treatment options. The advent of other TKIs and ICIs has greatly expanded therapeutic choices. Notably, trials such as IMbrave150 and HIMALAYA have reshaped the first-line treatment paradigm, introducing combinations like atezolizumab with bevacizumab and durvalumab with tremelimumab as preferred therapies [13,14,15]. These advancements have provided more effective alternatives by improving patient outcomes in terms of overall survival and progression-free survival. In addition to these combination therapies, several other systemic treatments are also approved for advanced HCC [16]. First-line options include monotherapies with durvalumab, lenvatinib, sorafenib, or pembrolizumab. For subsequent-line treatments, choices encompass cabozantinib, regorafenib, sorafenib, lenvatinib, pembrolizumab, or a combination of nivolumab and ipilimumab.

However, despite these advancements in treatment, the overall prognosis for patients with advanced HCC remains poor. A considerable portion of this patient population does not respond effectively to current therapies, a challenge evident in clinical trials such as IMbrave150 and HIMALAYA [14,15]. In these trials, which each included an arm receiving the current standard-of-care systemic therapies, only 27.3% and 20.1% of participants exhibited a confirmed objective radiological response, respectively. Furthermore, up to 20% of participants were refractory to the combination of atezolizumab and bevacizumab in the IMbrave150 trial, and up to 40% had progressive disease while receiving the combination of durvalumab and tremelimumab in the HIMALAYA trial. In addition to this variability in individual patient responses to systemic therapies, there is also a considerable rate of adverse events associated with these treatments, along with the high cost of these medications [17,18].

**Figure 1:**
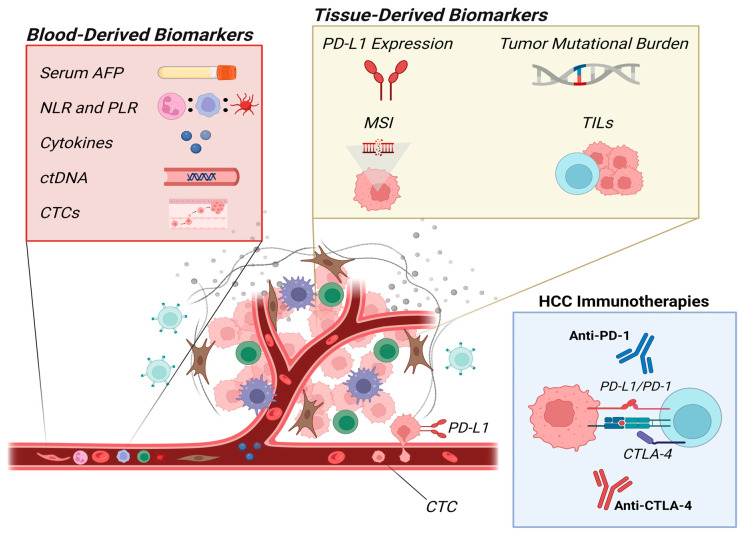
Overview of blood-derived and tissue-derived biomarkers in hepatocellular carcinoma immunotherapy. AFP: alpha-fetoprotein; NLR: neutrophil-to-lymphocyte ratio; PLR: platelet-to-lymphocyte ratio; ctDNA: circulating tumor DNA; CTC: circulating tumor cell; MSI: microsatellite instability; TIL: tumor-infiltrating lymphocyte.

Given these challenges, the identification and validation of biomarkers capable of predicting treatment response are crucial. Biomarkers hold promise for enhancing treatment efficacy, minimizing adverse effects, and optimizing healthcare resource allocation. As the field of HCC treatment continues to evolve, integrating predictive biomarkers into clinical practice could significantly improve patient outcomes by tailoring therapies to individual profiles. This review aims to provide valuable insights into the current body of evidence on the main studied biomarkers that may be applied to navigate the complexities of treating advanced HCC effectively in the era of expanding therapeutic options. Figure 1 and Table 1 provide a visual summary of the key biomarkers discussed in this manuscript and an overview of their associated clinical outcomes in the context of HCC immunotherapy, along with their measurement techniques and the challenges associated with the use of each biomarker.

## 2. Biomarkers

### 2.1. Blood-Derived Biomarkers

#### 2.1.1. Serum Alpha-Fetoprotein

Serum alpha-fetoprotein (AFP) is among the most extensively studied and widely used biomarkers in patients with HCC. Levels of this biomarker can be measured using globally available and affordable methods such as competitive radioimmunoassay and non-isotopic immunoassays on blood samples [19]. While AFP is found at a low level throughout life in normal adults, it is a major serum glycoprotein in the human fetus, mainly produced by the hepatic tissue and visceral endoderm of the yolk sac [20,21]. In adults, elevated AFP levels are predominantly indicative of a pathological condition and are associated with HCC and some subtypes of germ cell tumors [22,23]. In the context of HCC, studies have demonstrated that AFP plays a significant role in tumor progression by suppressing immune system activity and inhibiting T cell proliferation [22,24]. Additionally, AFP acts as a pro-proliferative agent, promoting the growth of cancerous tissues by hindering apoptotic signaling and modulating cytoplasmic pathways involved in cell survival and proliferation [25,26].

Elevated baseline AFP levels have been linked to both poorer prognosis in patients with HCC and diminished response to systemic treatment. A pooled analysis of 28 retrospective studies involving 3402 patients revealed that among those treated with ICIs, higher baseline AFP levels were associated with increased hazards of death (hazard ratio, HR, of 1.69; 95% confidence interval, CI, of 1.51–1.89) and disease progression or death (HR of 1.38; 95% CI of 1.19–1.61), as well as lower odds of achieving disease control (odds ratio, OR, of 0.44; 95% CI of 0.31–0.64) [27]. These relationships were consistent for the AFP cutoff value of 400 ng/mL, with those having AFP ≥ 400 ng/mL showing significantly shorter overall survival (OS) and progression-free survival (PFS), and a lower disease control rate (DCR), compared to those with AFP < 400 ng/mL. However, conflicting findings regarding the impact of baseline AFP levels on survival outcomes have been reported in phase III randomized clinical trials (RCTs) evaluating the efficacy and safety of systemic treatments for advanced HCC. In the IMbrave150 trial (comparing atezolizumab and bevacizumab to sorafenib) and the RATIONALE-301 trial (comparing tislelizumab to sorafenib), it was observed that OS was longer among those with AFP levels less than 400 ng/mL [14,28]. Conversely, in the HIMALAYA trial (comparing tremelimumab and durvalumab to sorafenib, and durvalumab alone to sorafenib), the CheckMate 459 trial (comparing nivolumab to sorafenib), and the LEAP-002 trial (comparing pembrolizumab and lenvatinib to lenvatinib alone), those with AFP levels greater than 400 ng/mL had lower hazards of death [15,29,30]. These discrepancies underline the complex role of AFP as a prognostic biomarker in HCC and suggest that the relationship between AFP levels and treatment outcomes may be influenced by factors such as the specific treatment regimen and patient heterogeneity, emphasizing the need for further studies.

Other than pretreatment levels of AFP, studies suggested a potential predictive role in outcomes for the changes in AFP levels following systemic treatment in patients with HCC. Most of those studies, however, evaluated systemic treatments such as chemotherapy and TKIs [31]. Additionally, it is critical to note that the definitions used for AFP response and the timing of its measurement varied across those studies. To investigate the relationship between changes in AFP levels and response to immunotherapy, a post hoc analysis was conducted using data from the GO30140 and IMbrave150 trials [14,32,33]. The GO30140 trial served as a training cohort to estimate appropriate AFP response cutoffs and time points. These estimates were then validated using updated efficacy data from the atezolizumab and bevacizumab arm of the IMbrave150 trial. Two AFP cutoffs from baseline at 6 weeks were considered: a ≥75% decrease, which distinguished responders from non-responders, and a ≤10% increase, which identified disease control. Both AFP cutoffs were found to be associated with longer OS and PFS. Specifically, a reduction of AFP by ≥75% demonstrated a sensitivity of 0.71 and 0.59, and a specificity of 0.91 and 0.86, in the GO30140 and IMbrave150 trials, respectively, for identifying responders. Meanwhile, a ≤10% increase in AFP showed a sensitivity of 0.89 and 0.77, and a specificity of 1.00 and 0.44, in the respective trials. Similar findings were reported in a meta-analysis of retrospective studies, where significant relationships between early AFP response following ICI treatment and longer OS and PFS, as well as higher objective response rate (ORR) and DCR, were observed [27]. These findings underscore the value of AFP as a dynamic biomarker for assessing treatment response and guiding clinical decisions in HCC, especially in the context of immunotherapy.

#### 2.1.2. Neutrophil-to-Lymphocyte Ratio and Platelet-to-Lymphocyte Ratio

The neutrophil-to-lymphocyte ratio (NLR) and platelet-to-lymphocyte ratio (PLR) are emerging as valuable prognostic markers in various malignancies, including HCC. These ratios are computed by dividing the absolute neutrophil count and absolute platelet count by absolute lymphocyte count and can be easily derived from routine complete blood count results. These blood cells, neutrophils and platelets, could significantly contribute to tumoral growth and progression by producing and releasing various inflammatory cytokines and growth factors such as vascular endothelial growth factor, which facilitates tumor vascularization [34,35]. On the other hand, lymphocytes play critical roles in the development of adaptive antitumor immunity. Therefore, these ratios may reflect the balance between the proinflammatory activity of neutrophils and platelets and the antitumoral activity of lymphocytes, offering insights into the systemic inflammatory response and its influence on cancer progression.

Elevated NLR and PLR are, therefore, indicative of a pro-tumoral environment and are consistently linked to poorer survival outcomes and treatment responses in patients with HCC. A study involving 100 patients with unresectable HCC who received atezolizumab and bevacizumab as their first-line therapy found that an NLR cut-off of 3.20, optimized via receiver operating characteristic curves, was the most significant independent predictor of ORR and PFS, as well as a significant predictor of OS [36]. Similarly, a meta-analysis of observational studies revealed that elevated NLR and PLR correlate with worse OS and PFS among patients receiving ICIs for HCC [27]. A recent multicenter study across the United States, Europe, and Asia with 296 patients with unresectable HCC treated with atezolizumab and bevacizumab further validated these findings [37]. It demonstrated that patients with an NLR greater than 5 experienced significantly poorer survival outcomes, with OS at 9.4 months compared to 16.8 months and PFS at 4.9 months versus 7.58 months. Additionally, a PLR ≥ 300 was associated with shorter OS (9.4 months vs. 15.7 months) and PFS (3.4 months vs. 7.1 months). In multivariable analysis, only NLR > 5 remained significantly associated with OS, while neither NLR nor PLR independently predicted PFS. Moreover, no significant relationship was found between baseline NLR or PLR and radiological responses.

In addition to baseline NLR and PLR, post-treatment NLR and PLR also offer substantial predictive value. A study of 203 patients with advanced HCC treated with nivolumab found that both pre- and post-treatment NLR (with a cutoff of 5) and PLR tertiles were strongly associated with survival outcomes [38]. Multivariable analysis revealed a more robust predictive value for post-treatment NLR and PLR. Notably, a composite variable of post-treatment NLR and PLR was associated with an eightfold increase in the risk of death, underscoring the combined prognostic significance of these markers. Furthermore, studies indicate that these ratios can also predict the occurrence of immune-related adverse events in patients with cancer undergoing treatment with ICIs. A systematic review and meta-analysis of 47 studies with 11,491 cancer patients receiving ICIs showed that a lower pre-treatment NLR was associated with a higher overall rate of immune-related adverse events, whereas higher baseline NLR was more frequently linked to immune-related liver injury [39]. Therefore, while lower NLR and PLR are associated with better treatment efficacy and improved survival outcomes, they may also signal a higher likelihood of immune-related adverse events. This presents a challenge in their application, as using these ratios to predict treatment efficacy must be carefully balanced against the potential for increased immune-related adverse events. Additionally, the absence of standardized cutoffs across studies complicates their clinical use, necessitating further research to establish consistent and reliable thresholds. This will enhance their utility in optimizing treatment strategies for HCC while managing associated risks.

#### 2.1.3. Cytokines

Cytokines, pivotal signaling proteins secreted by tumoral cells, their adjacent nontumoral cells, and immune cells, orchestrate intricate immune responses. These soluble signaling mediators play critical roles in the modulation of immune cell functions and their dynamic interaction with malignancies such as HCC [40,41,42]. Their levels could readily be measured through various laboratory techniques such as bioassays, immunoassays, and flow cytometry [40]. Emerging evidence underscores that cytokine profiles may serve as vital biomarkers, offering valuable insights into the treatment efficacy of immunotherapy in patients with advanced HCC. This section explores the predictive role of various cytokines that have been studied in the literature in optimizing the therapeutic outcomes of ICIs in the management of HCC.

The pro-inflammatory cytokine interleukin-6 (IL-6) has been extensively studied relative to other interleukins due to its multifaceted role in immune modulation and tumor progression. Recent evidence underscores that elevated IL-6 levels contribute to the establishment of an immunosuppressive tumor microenvironment and are indicative of poor prognosis and resistance to ICIs. In addition, experimental studies in a variety of murine tumor models, including HCC models, have demonstrated the potential of targeting IL-6 in conjunction with PD-1/PD-L1 blockade to reverse ICI resistance [43,44,45]. A prospective cohort study in Korea involving 165 patients with advanced HCC treated with atezolizumab and bevacizumab revealed that serum IL-6 concentrations were markedly higher in participants who did not experience any clinical benefits within six months of systemic therapy initiation [46]. Furthermore, the study found that patients with elevated baseline levels of this cytokine exhibited lower ORR and poorer OS and PFS compared to those with lower IL-6 levels. Multivariable analysis identified high baseline IL-6 as the most important factor correlating with adverse PFS and OS outcomes. Additionally, comparative analyses of peripheral blood cytotoxic T lymphocytes’ production profile and intratumoral immune cell subsets demonstrated that patients with elevated IL-6 levels exhibited reduced interferon-gamma (IFN-γ) and tumor necrosis factor-alpha (TNF-α) secretion and a non-T cell-inflamed immunosuppressive tumor microenvironment, respectively. These findings are consistent with other reports on the predictive role of IL-6, another prospective cohort study that identified IL-6 as an independent prognostic factor for disease progression, with high IL-6 levels also predicting shorter PFS and OS [47,48].

In addition to IL-6, IL-8 has been shown to play significant roles in predicting the efficacy of ICIs in advanced HCC, primarily through its role in promoting resistance via recruitment of myeloid-derived suppressor cells [49,50,51]. A retrospective study involving 80 patients with advanced HCC receiving ICIs demonstrated significant elevations in IL-8 levels among those failing to achieve disease control after the initial dose of immunotherapy and prior to the second dose [52]. In contrast, no substantial changes in IL-8 levels were noted among responders. Moreover, these early alterations in IL-8 levels correlated significantly with response to the treatment, yielding an area under the curve (AUC) of 0.81, with a cutoff of an 8.1% increase in IL-8 providing a promising sensitivity and specificity of 70.6% and 87.0%, respectively. Beyond its association with therapeutic response, elevated IL-8 levels emerged as an independent prognostic factor for reduced OS and PFS. This observation is consistent with findings from a meta-analysis assessing the predictive utility of IL-8 in patients with cancer undergoing ICI therapy, though advanced HCC was not specifically included in the analysis [53]. The meta-analysis, encompassing over 3000 patients, indicated that, among those with elevated IL-8 levels, there were significantly lower ORR, OS, and PFS.

Transforming Growth Factor-beta (TGF-β) also holds promise as a predictive biomarker for immunotherapy efficacy in advanced HCC. This multifunctional cytokine exerts broad immunomodulatory effects, particularly influencing the function and proliferation of the regulatory T cells subset, thereby suppressing cytotoxic T cell activity [54,55,56]. TGF-β is the sole cytokine that had its role as a biomarker of response to the treatment evaluated in a Phase II clinical trial examining the efficacy of ICIs among participants with advanced HCC [57]. In this trial, 28 patients with advanced HCC were treated with pembrolizumab. Results of this study indicated that responders had significantly lower levels of TGF-β. A threshold value of 200 pg/mL, derived from near median values, was established for TGF-β levels, and it was revealed that those with lower than this threshold level of TGF-β had significantly longer OS (over 25 months versus 7 months) and PFS (over 25 months versus 2 months).

Beyond IL-6, IL-8, and TGF-β, other cytokines may also potentially influence the efficacy of ICIs in patients with advanced HCC. Although these cytokines have not been studied in clinical studies specifically for patients with advanced HCC, their roles have been evaluated in other malignancies such as melanoma, advanced renal cell carcinoma, and non-small cell lung cancer under immunotherapy regimens. Elevated levels of cytokines such as IL-2, IL-4, IL-10, IL-12, IFN-γ, and TNF-α have been associated with altered responses to ICIs in these cancers [58,59,60,61,62,63]. These observations, combined with in vitro and mechanistic studies that highlight the critical role of these biomarkers in modulating the tumor microenvironment and immune responses in patients with various stages of HCC, suggest their broader relevance in predicting immunotherapy outcomes in this patient population [64,65]. This underscores the potential for a more comprehensive cytokine profile to serve as a predictive tool for immunotherapeutic efficacy in HCC.

#### 2.1.4. Circulating Tumor DNA

**Figure 2:**
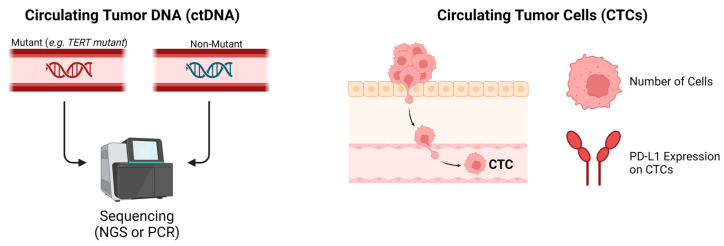
Circulating tumor DNA (CtDNA) and circulating tumor cells (CTCs) as liquid biopsy blood-derived biomarkers for immunotherapy response in hepatocellular carcinoma.

Circulating tumor DNA (ctDNA) consists of DNA fragments released into the bloodstream from tumor cells through metabolic secretion, apoptosis, or necrosis. As a subset of circulating free DNA (cfDNA), ctDNA has garnered considerable interest in current HCC research due to its minimally invasive sampling advantages (Figure 2). Additionally, ctDNA shows potential for early detection of various cancers, including HCC, and serves as a prognostic biomarker for overall patient outcomes and as a predictor of response not only to immunotherapy but also to other therapeutic options such as radiotherapy [66,67,68,69,70,71]. Despite this promise, the body of evidence supporting the predictive role of ctDNA in assessing responses to ICIs in advanced HCC remains limited.

Existing studies have not only examined the correlation between ctDNA levels and clinical outcomes but have also characterized mutations within these DNA fragments to evaluate their prognostic significance. In a multicenter prospective cohort study of 85 patients with advanced HCC undergoing treatment with atezolizumab and bevacizumab, baseline cfDNA levels were measured and their association with clinical outcomes were assessed [72]. The study found that patients with low cfDNA levels exhibited significantly higher ORR, and longer OS and PFS, compared to those with elevated cfDNA levels. Beyond these quantitative analyses, the study sequenced 25 HCC frequently mutated genes, identifying TERT promoter mutations as the most common within the patient cohort. Notably, the presence of mutations in the TERT promoter was associated with superior OS, highlighting their potential role in prognostication. Complementing these findings, Hsu et al. investigated the dynamics of this biomarker and evaluated the association between changes in ctDNA levels and treatment response among 47 patients with advanced HCC receiving first-line therapy with atezolizumab and bevacizumab. A transition from positive to undetectable ctDNA levels was observed exclusively in patients achieving disease control, with higher rates among those with a complete response (70%) and partial response (27%), compared to patients with stable disease (9%). Additionally, patients experiencing this change had significantly longer PFS [73].

#### 2.1.5. Circulating Tumor Cells

Circulating tumor cells (CTCs) provide another valuable source of information obtained through liquid biopsy, potentially aiding in the treatment planning for patients with advanced HCC. These tumor cells enter the bloodstream after detachment from the primary tumor or its metastatic sites, with a fraction of them colonizing distant organs and initiating the process of metastasis [74,75] (Figure 2). The close correlation between these cells, tumor burden, the extent of tumor invasion, the propensity for hematogenous spread, and their potential to predict poor outcomes in various cancers, suggest they may also serve as biomarkers for predicting responsiveness to immunotherapy [76,77,78,79,80].

Currently, investigations into the relevance of CTCs and response to the treatment predominantly focus on their relevance in surgical interventions such as liver resection, rather than immunotherapeutic approaches [81]. In a small cohort of 10 patients with advanced HCC treated with PD-1 inhibitors, the presence of PD-L1-expressing CTCs (PD-L1+ CTCs) correlated with treatment response: five out of six patients with PD-L1+ CTCs achieved disease control, contrasting with no response among the four patients lacking PD-L1 expression [82]. Beyond this, the predictive role of PD-L1+ CTC counts has also been examined. In a single-arm trial of 47 patients with advanced HCC undergoing triple therapy with a PD-1 inhibitor, a TKI, and conformal radiation therapy, those with fewer than 2 PD-L1+ CTCs at baseline exhibited superior efficacy, including higher ORR and longer OS [83]. Additionally, it was found that PD-L1+ CTC counts decreased upon the start of the triple therapy in responders while remaining unchanged in those with progressive disease. This observation was corroborated by another single-arm trial involving 124 participants with unresectable HCC receiving immunotherapy, where individuals with fewer than 2 PD-L1+ CTCs demonstrated longer PFS and OS [84]. Additionally, a recent study by Nosaka et al. prospectively assessed 22 patients treated with atezolizumab and bevacizumab and 24 patients treated with lenvatinib [85]. Among those undergoing treatment with atezolizumab and bevacizumab, patients with higher PD-L1 mRNA expression in CTCs exhibited superior ORR, PFS, and OS. PD-L1 mRNA expression levels decreased among responders, while patients with both initial and subsequent progressive disease experienced a rise in expression levels. Conversely, among participants treated with lenvatinib monotherapy, no association was observed between PD-L1 mRNA expression levels and clinical outcomes, nor was there a significant change in expression levels during the course of treatment.

Therefore, evaluating and monitoring these tumor-derived genomic content and cells, ctDNA and CTC, through non-invasive techniques holds promise for advanced HCC management, yet their validation in larger cohorts is essential to confirm their predictive role. It is also noteworthy to address the formidable challenges in the detection of these cells, stemming from their low abundance in peripheral blood, variable expression of adhesion molecules, and the diversity of physical and biological methods employed for isolation [86,87,88,89]. Furthermore, certain measurement techniques interact with surface molecular evaluations of these cells, underscoring the imperative for methodological standardization in quantifying circulating tumor cells.

### 2.2. Tissue-Derived Biomarkers

#### 2.2.1. PD-L1 Expression

PD-L1 expression is the most commonly studied tissue-derived biomarker in clinical trials involving patients with advanced HCC. This crucial ligand engages with the coinhibitory PD-1 receptor located on the surface of various infiltrating immune cells within the tumor microenvironment, such as T and B lymphocytes and dendritic cells [90]. The engagement of PD-L1 with PD-1 results in the suppression of immune cell activation and proliferation, thereby enabling the tumor to evade antitumor immunity [91,92]. Consequently, elevated PD-L1 expression may suggest that ICIs targeting PD-L1 may effectively counteract the tumor’s immunosuppressive tactics. PD-L1 expression is typically assessed through immunohistochemistry on cancerous tumor tissue biopsies, either solely on the tumor cells or on both the tumor cells and the adjacent infiltrated immune cells, providing a quantifiable measure of PD-L1 protein levels as a tumor proportional score (TPS) or combined positive score (CPS), respectively [93,94].

The role of PD-L1 expression as a predictive biomarker for ICIs and its clinical utility among patients with advanced HCC continues to be debated, despite a substantial body of supporting evidence. Findings from the phase II non-randomized KEYNOTE-224 trial, evaluating pembrolizumab monotherapy in patients previously treated with sorafenib, suggest an association between PD-L1 expression and treatment response [95]. Among the 52 participants with available PD-L1 expression data, the study observed significantly higher ORR and PFS in those with PD-L1 expression ≥ 1%, as determined by the CPS, whereas the TPS did not yield a similar correlation. Similarly, in the dose-expansion phase of the multicenter CheckMate 040 trial, which investigated the PD-1 inhibitor nivolumab in patients with advanced HCC, CPS at 1% was not associated with having an objective response [96]. A systematic review and meta-analysis on PD-L1 expression in this patient population undergoing immunotherapy, encompassing 11 clinical trials and 1330 patients with advanced HCC, found that PD-L1 expression was associated with a significantly higher ORR (26% vs. 18%) but not DCR [97]. Another systematic review and meta-analysis revealed that both CPS and TPS were associated with higher ORR [98].

The utility of PD-L1 expression as a biomarker for predicting response to immunotherapy in HCC also faces other significant challenges. Firstly, while immunohistochemistry remains the primary method for assessing PD-L1 expression, the various techniques used to detect PD-L1—including different assays, antibody clones, staining platforms, and scoring systems such as TPS and CPS—along with varying cut-off points, complicate the consistent assessment of PD-L1 levels [94]. This variability can potentially affect the generalizability and predictive value of PD-L1 expression as a biomarker. Standardization across these parameters is crucial to achieve uniformity and ensure reliable clinical interpretation of PD-L1 status in treatment decision-making. Additionally, the subjective nature of interpretation contributes to variability in PD-L1 quantification, with studies demonstrating significant discrepancies between pathologists performing the assessments [99]. In addition, studies have shown that specimen conditions significantly influence PD-L1 expression. Factors such as sample age and the method of sampling exert notable effects on PD-L1 levels [100,101,102]; in several clinical trials involving patients with advanced HCC, PD-L1 expression was retrospectively reviewed, potentially contributing to the discussed inconsistencies in the reported findings. Lastly, it has been reported that the expression of PD-L1 could be influenced by several factors, including prior treatments such as sorafenib [103,104]. This treatment-induced variability in PD-L1 expression poses a challenge in accurately assessing its prognostic and predictive value, especially in patients who may undergo multiple lines of treatment with various systemic therapies, as the timing of biopsy relative to prior therapies can lead to significant fluctuations in PD-L1 status.

#### 2.2.2. Tumor Mutational Burden

Tumor mutational burden (TMB) quantifies the number of somatic mutations per megabase within the sequenced genome of the tumor cells. Utilizing a comprehensive genomic profiling approach across 315 cancer-related genes, Ang et al. reported a median TMB of four mutations per megabase in a cohort of 755 participants with advanced HCC. Of these, 188 and six participants exhibited intermediate and high TMB, defined in this study as having 6–19 and ≥20 mutations per megabase, respectively [105]. Elevated TMB levels are generally indicative of an increased potential in the production of neoantigens and thereby enhanced tumor immunogenicity. This heightened immunogenicity can facilitate the recognition of these antigens by the immune cells, which may result in a more favorable efficacy with ICIs [106].

Although most studies exploring TMB’s predictive value have focused on other cancers such as melanoma, non-small cell lung cancer, and urothelial carcinoma, recent research suggests a relevance in HCC, despite inconsistent findings [107,108,109,110]. In a Phase I trial on the efficacy and safety of a combination of camrelizumab and apatinib, 18 participants with advanced HCC and 25 participants with advanced gastric or esophagogastric junction cancer were involved [111]; however, data on TMB status was available for only 18 participants. In this subgroup, it was found that TMB status significantly predicted response to the combination treatment and, while participants with higher TMB exhibited PFS, this did not reach statistical significance. In a secondary analysis of the data of 76 patients from the GO30140 trial and 130 patients from the IMbrave150 trial, all of whom had advanced HCC and received treatment with atezolizumab and bevacizumab, the association between baseline TMB and clinical outcomes were assessed [112]. In the GO30140 trial, higher ORR was observed in the highest TMB tertile, yet no significant differences in PFS were found between the tertiles. Moreover, the study found that TMB tertiles did not correlate with objective responses, PFS, or OS in the IMbrave150 trial. This absence of a predictive role for TMB was also reported in a retrospective study of 99 patients with advanced HCC treated either with nivolumab or pembrolizumab, where no association between TMB and both PFS and ORR was observed [113]. These inconsistencies, compounded by variations in the sample acquisition techniques, limited availability, high costs, significant time requirements, and the lack of standardized sequencing protocols, constrain the current clinical utility of TMB as a predictive biomarker at present. Additionally, the low prevalence of high TMB among patients with advanced HCC suggests that TMB alone may not serve as an ideal predictive marker for ICI response, owing to the limited eligible participant pool. Consequently, further studies are warranted to more comprehensively evaluate the predictive capacity of TMB in the context of immunotherapy for advanced HCC.

#### 2.2.3. Microsatellite Instability

Microsatellite instability (MSI), characterized by the presence of frequent insertions or deletions within repetitive DNA sequences, predominantly arises from deficiencies in the DNA mismatch repair (MMR) system [114]. Analogous to TMB, these instabilities in the microsatellite sequences induce a heightened mutation rate, thereby promoting the generation of neoantigens, which enhances tumor immunogenicity and facilitates earlier immune recognition and boosts the immune response [115,116,117]. The promising role observed for MSI in predicting responses to ICIs across a wide range of malignancies has established its potential as a pan-cancer biomarker, raising expectations for its applicability in other unstudied malignancies, including advanced HCC [118,119]. Reflecting this, the U.S. Food and Drug Administration (FDA) granted accelerated approval to pembrolizumab on 23 May 2017 for patients with nonresectable or metastatic solid tumors exhibiting high MSI (MSI-H) or deficient MMR (dMMR) who have not achieved disease control with prior lines of treatment or have no other satisfactory treatment option [120,121]. This landmark approval was the first to recognize a therapy for patients with cancer based on a genetic anomaly rather than the tumor’s location. In the pivotal trials underpinning this approval, KEYNOTE-012, KEYNOTE-028, KEYNOTE-016, KEYNOTE-158, and KEYNOTE-164, 149 out of 415 patients were MSI-H/dMMR, with over 60% having colorectal cancer, while the remainder spanned 14 other tumor types. Consequently, while this approval extends to patients with advanced HCC, the specific predictive role of MSI in HCC remains underexplored, with evidence limited to a few case reports rather than robust dedicated observational or interventional studies [122,123]. The clinical utility of MSI as a biomarker in advanced HCC is further constrained by its low prevalence in this patient population, with studies indicating MSI-H/dMMR rates ranging from negligible to less than 3% [124]. Therefore, even if future studies validate MSI-H/dMMR’s predictive value in patients with advanced HCC, its practical application should be considered more as an inclusion criterion due to the low incidence among patients, rather than an exclusion criterion. Additionally, the assessment of MSI/MMR status presents significant challenges due to methodological variability, including differences between immunohistochemistry and polymerase chain reaction assays, as well as discrepancies between local and centralized testing approaches, which complicate standardization and can impact the reliability and comparability of MSI/MMR status evaluations across studies and clinical settings.

#### 2.2.4. Tumor-Infiltrating Lymphocytes

Tumor-infiltrating lymphocytes (TILs) represent another significant class of biomarkers that reflect the immune response dynamics within the tumor microenvironment in various cancers, including HCC. These infiltrated lymphocytes, situated either within the tumor or adjacent to tumor cells, are comprised of various subsets of T cells, notably cytotoxic T cells and helper T cells. TILs are the main cells involved in exerting antitumor immunity through mechanisms such as direct tumor cell recognition and attack, as well as cytokine secretion that modulates each and every aspect of the immune response [125]. The presence, activity, density, and composition of TILs are indicative of the host’s capacity to mount an effective immune defense against the tumor. Additionally, a substantial body of evidence demonstrates that characteristics of TILs are significantly associated with clinical outcomes and response to immunotherapy in patients with advanced HCC.

A secondary analysis of the CheckMate 040 Phase I/II trial, where participants with advanced HCC received nivolumab monotherapy, found that increased presence of T cells expressing CD3+, CD4+, CD8+, or FOXP3+, as well as M2 macrophages (CD68+, CD163+), was not associated with objective responses or OS [126]. In data from another Phase I trial administrating a combination of sintilimab and a bevacizumab biosimilar for patients with advanced HCC, the profile of TILs was analyzed to explore its association with clinical outcomes [127]. Similar to the findings in the CheckMate 040 trial, no significant relationship was identified between the efficacy measures and the density or percentage of T cells expressing CD3+, CD4+, CD8+, and FoxP3+, CD20+ cells, or M2 macrophages in either the tumor or stromal areas. However, this analysis revealed that increased levels of total macrophages (CD68+) in the stromal area and natural killer cells in the tumor area correlated with clinical benefits. Notably, among the different TIL subsets, only the density of M1 macrophages (CD68+, CD163-) demonstrated a positive association with PFS and OS. Zhu et al. also utilized the baseline data on TILs from two clinical trials, the GO30140 and the IMBrave150 trials. They found that higher densities of CD3+ and CD8+ cells in the tumor area predicted a favorable response to the combination of atezolizumab and bevacizumab in the GO30140 trial [112]. In addition, in the IMBrave150 trial, they found that increased intratumoral CD8+ cell density was linked to a significantly lower hazard of death (0.29; 95% CI of 0.14–0.61) and a reduced risk of disease progression or death (0.54; 95% CI of 0.29–1.00).

Beyond the results from the discussed clinical trials, additional reports underscore the association between various TIL subsets and clinical outcomes of immunotherapy in patients with advanced HCC [128,129,130,131]. Despite these promising associations, challenges remain, with particularly the heterogeneity of findings across studies and the diversity of TIL populations being implicated. This variability underscores the need for a more nuanced understanding of the relationship between different immune cell subsets and HCC prognosis. Enhancing our grasp of these dynamics, especially with the advent of cutting-edge methods like single-cell RNA sequencing [132,133,134], may not only enable the better characterization of these novel biomarkers for predicting responses to ICIs in advanced HCC and integrate them into the current standard-of-care but also any unveil new therapeutic targets, fostering the development and application of innovative ICIs.

## 3. Current Challenges and Future Directions

While the advent of immunotherapies for patients with advanced HCC revolutionized the systemic treatment options for these patients and became the current standard-of-care, numerous challenges remain that impede its broader application and efficacy. HCC exhibits notable diversity in its genetic, molecular, and cellular compositions among individuals, as well as within the same tumor of a single patient due to intratumoral heterogeneity, even in uninodular cases [135,136]. This variability makes it difficult to identify to develop standardized treatment protocols and universal biomarkers applicable across all patient populations. In addition, due to this intertumoral heterogeneity, different regions may exhibit distinct genetic profiles and tumor microenvironments, complicating the reliability and applicability of biomarkers such as TILs and PD-L1 expression [137,138,139]. Variability in sample acquisition, processing techniques, and biomarker assessment methodologies also poses significant challenges in HCC research and clinical practice for some of these biomarkers, hindering the generalization of the findings and the integration of results across diverse studies, thereby limiting the ability to draw unified conclusions. Standardizing protocols for biomarker assessment could be beneficial for ensuring the reliability and reproducibility of findings across different studies and settings. Additionally, the high cost and limited accessibility of biomarkers like TMB, ctDNA, and CTC present formidable barriers to their widespread adoption, particularly in resource-constrained healthcare settings. Some of these biomarkers have shown potential through dynamic and serial measurements, as discussed in this article, further complicating their utility due to the associated affordability issues for patients.

Another critical challenge in the existing literature concerning the predictive role of biomarkers in immunotherapy efficacy—applicable to nearly all discussed biomarkers—is the predominance of observational or single-arm trials. In those studies, immunotherapy is uniformly administered to all participants, making these biomarkers primarily prognostic rather than predictive. The absence of an untreated control group precludes the calculation of the biomarker’s interaction with treatment, limiting our ability to discern their predictive value [140,141]. Moving forward, prioritizing studies that include untreated control groups alongside immunotherapy-treated cohorts, preferably through RCTs, will be essential. RCTs with biomarker-stratified randomization allow for prospective evaluation of biomarkers in predicting treatment response, validating their utility, and providing robust evidence for clinical decision-making.

Additionally, a notable challenge across many studies exploring biomarkers for predicting immunotherapy efficacy is the inadequate sample sizes. Many of those studies have been hindered by small cohorts, limiting statistical power and the generalizability of findings, warranting larger, well-powered cohorts with diverse patient populations in future research efforts. In addition, the majority of those studies focused on whether there is a significant association between biomarker levels and response to immunotherapy. Furthermore, among those studies that reported such an association, only some of them have provided the effect size of this association. Furthermore, detailed performance metrics like sensitivity and specificity, which are crucial for assessing the practical utility of biomarkers in a clinical setting, are frequently absent in the current studies.

Lastly, one important point to note is that the treatment plan for complex conditions such as advanced HCC, which requires comprehensive multidisciplinary management, cannot rely solely on a single biomarker. There are multiple aspects that must be considered, and current studies indicate that single biomarkers often show suboptimal sensitivity and specificity. Incorporating additional data, such as the clinical characteristics of patients—including the etiology of HCC, liver function, and performance status—as well as imaging features, can offer unique insights into disease biology and treatment response [4,142,143]. Furthermore, integrating multi-omics approaches with data sets of different omic groups including genomics, transcriptomics, proteomics, and metabolomics, and developing gene signatures, have shown robust and promising results in recent studies on immunotherapy response among patients with advanced HCC [144,145,146]. These approaches allow for a more comprehensive understanding of tumor behavior and patient-specific factors, facilitating more personalized and effective treatment strategies. By combining multiple biomarkers and data sources, clinicians can better stratify patients, predict therapeutic responses, and monitor treatment efficacy over time, thus advancing the precision and effectiveness of therapeutic interventions in HCC management.

## Figures and Tables

**Table 1:** Measurement methods and challenges of biomarkers for immunotherapy outcomes in hepatocellular carcinoma.

Biomarker	Measurement Method	Limitations
Blood-derived biomarkers		
Serum alpha-fetoprotein	Radio- and fluorescent-immunoassay techniques	Lacks sensitivity; not always indicative of HCC
Neutrophil-to-lymphocyte ratio and platelet-to-lymphocyte ratio	Complete blood count	Standardized cutoffs needed; varies between studies
Cytokines	Immunoassays, multiplex assays	More HCC-specific clinical trials are needed for definitive conclusions
Circulating tumor DNA	Next-generation sequencing, digital PCR	Challenges in isolating ctDNA; not consistently reliable as a biomarker
Circulating tumor cells	Filtration, electrophoresis, antigen-antibody binding	Low levels in early-stage HCC; conflicting study results
Tissue-derived biomarkers		
PD-L1 expression	Immunohistochemistry, cancerous tumor tissue biopsies	Controversial due to mixed results; not effective for all treatments
Tumor mutational burden	Next-generation sequencing	Lacks consistency; independent from PD-L1 expression
Microsatellite instability	Polymerase chain reaction	MSI-H is rare in HCC, limiting its standalone use
Tumor-infiltrating lymphocytes	Immunohistochemistry, single-cell RNA sequencing	Inconsistent data; presence varies among patients

## Data Availability

Not applicable.

## References

[1] Sung H., Ferlay J., Siegel R.L., Laversanne M., Soerjomataram I., Jemal A., Bray F. (2021). Global Cancer Statistics 2020: GLOBOCAN Estimates of Incidence and Mortality Worldwide for 36 Cancers in 185 Countries. CA Cancer J. Clin..

[2] Rumgay H., Arnold M., Ferlay J., Lesi O., Cabasag C.J., Vignat J., Laversanne M., McGlynn K.A., Soerjomataram I. (2022). Global burden of primary liver cancer in 2020 and predictions to 2040. J. Hepatol..

[3] Janevska D., Chaloska-Ivanova V., Janevski V. (2015). Hepatocellular Carcinoma: Risk Factors, Diagnosis and Treatment. Open Access Maced. J. Med. Sci..

[4] Shi Y., Taherifard E., Saeed A., Saeed A. (2024). MASLD-Related HCC: A Comprehensive Review of the Trends, Pathophysiology, Tumor Microenvironment, Surveillance, and Treatment Options. Curr. Issues Mol. Biol..

[5] Edoardo P., Eleonora D.M. (2024). Progression of liver disease and associated risk of hepatocellular carcinoma. Hepatoma Res..

[6] Shannon A.H., Ruff S.M., Pawlik T.M. (2022). Expert Insights on Current Treatments for Hepatocellular Carcinoma: Clinical and Molecular Approaches and Bottlenecks to Progress. J. Hepatocell. Carcinoma.

[7] Seif El Dahan K., Reczek A., Daher D., Rich N.E., Yang J.D., Hsiehchen D., Zhu H., Patel M.S., Bayona Molano M.d.P., Sanford N. (2023). Multidisciplinary care for patients with HCC: A systematic review and meta-analysis. Hepatol. Commun..

[8] Byrd K., Alqahtani S., Yopp A.C., Singal A.G. (2021). Role of Multidisciplinary Care in the Management of Hepatocellular Carcinoma. Semin. Liver Dis..

[9] Mandlik D.S., Mandlik S.K., Choudhary H.B. (2023). Immunotherapy for hepatocellular carcinoma: Current status and future perspectives. World J. Gastroenterol..

[10] Fan Y., Xue H., Zheng H. (2022). Systemic Therapy for Hepatocellular Carcinoma: Current Updates and Outlook. J. Hepatocell. Carcinoma.

[11] van Bömmel F., Berg T., Lordick F. (2023). Immune checkpoint inhibition (ICI) in current systemic therapies for hepatocellular carcinoma (HCC). ESMO Gastrointest. Oncol..

[12] Hartmann J.T., Haap M., Kopp H.G., Lipp H.P. (2009). Tyrosine kinase inhibitors—A review on pharmacology, metabolism and side effects. Curr. Drug Metab..

[13] Gordan J.D., Kennedy E.B., Abou-Alfa G.K., Beal E., Finn R.S., Gade T.P., Goff L., Gupta S., Guy J., Hoang H.T. (2024). Systemic Therapy for Advanced Hepatocellular Carcinoma: ASCO Guideline Update. J. Clin. Oncol..

[14] Finn R.S., Qin S., Ikeda M., Galle P.R., Ducreux M., Kim T.Y., Kudo M., Breder V., Merle P., Kaseb A.O. (2020). Atezolizumab plus Bevacizumab in Unresectable Hepatocellular Carcinoma. N. Engl. J. Med..

[15] Abou-Alfa G.K., Lau G., Kudo M., Chan S.L., Kelley R.K., Furuse J., Sukeepaisarnjaroen W., Kang Y.K., Van Dao T., De Toni E.N. (2022). Tremelimumab plus Durvalumab in Unresectable Hepatocellular Carcinoma. NEJM Evid..

[16] National Comprehensive Cancer Network (2024). Hepatocellular Carcinoma. https://www.nccn.org/professionals/physician_gls/pdf/hcc.pdf.

[17] Martins F., Sofiya L., Sykiotis G.P., Lamine F., Maillard M., Fraga M., Shabafrouz K., Ribi C., Cairoli A., Guex-Crosier Y. (2019). Adverse effects of immune-checkpoint inhibitors: Epidemiology, management and surveillance. Nat. Rev. Clin. Oncol..

[18] Kelly Z.R., Davar D. (2019). The financial and physical toxicity of immune checkpoint inhibitors in cancer. ASCO Daily News.

[19] Premnath S.M., Zubair M. (2023). Laboratory Evaluation of Tumor Biomarkers.

[20] Lazarevich N.L. (2000). Molecular mechanisms of alpha-fetoprotein gene expression. Biochemistry.

[21] Ball D., Rose E., Alpert E. (1992). Alpha-fetoprotein levels in normal adults. Am. J. Med. Sci..

[22] Galle P.R., Foerster F., Kudo M., Chan S.L., Llovet J.M., Qin S., Schelman W.R., Chintharlapalli S., Abada P.B., Sherman M. (2019). Biology and significance of alpha-fetoprotein in hepatocellular carcinoma. Liver Int..

[23] Jezierska M., Gawrychowska A., Stefanowicz J. (2022). Diagnostic, Prognostic and Predictive Markers in Pediatric Germ Cell Tumors-Past, Present and Future. Diagnostics.

[24] Terentiev A.A., Moldogazieva N.T. (2013). Alpha-fetoprotein: A renaissance. Tumour Biol..

[25] Lin B., Zhu M., Wang W., Li W., Dong X., Chen Y., Lu Y., Guo J., Li M. (2017). Structural basis for alpha fetoprotein-mediated inhibition of caspase-3 activity in hepatocellular carcinoma cells. Int. J. Cancer.

[26] Wang X.W., Xu B. (1998). Stimulation of tumor-cell growth by alpha-fetoprotein. Int. J. Cancer.

[27] Zhang L., Feng J., Kuang T., Chai D., Qiu Z., Deng W., Dong K., Zhao K., Wang W. (2023). Blood biomarkers predict outcomes in patients with hepatocellular carcinoma treated with immune checkpoint Inhibitors: A pooled analysis of 44 retrospective sudies. Int. Immunopharmacol..

[28] Qin S., Kudo M., Meyer T., Bai Y., Guo Y., Meng Z., Satoh T., Marino D., Assenat E., Li S. (2023). Tislelizumab vs Sorafenib as First-Line Treatment for Unresectable Hepatocellular Carcinoma: A Phase 3 Randomized Clinical Trial. JAMA Oncol..

[29] Llovet J.M., Kudo M., Merle P., Meyer T., Qin S., Ikeda M., Xu R., Edeline J., Ryoo B.Y., Ren Z. (2023). Lenvatinib plus pembrolizumab versus lenvatinib plus placebo for advanced hepatocellular carcinoma (LEAP-002): A randomised, double-blind, phase 3 trial. Lancet Oncol..

[30] Yau T., Park J.W., Finn R.S., Cheng A.L., Mathurin P., Edeline J., Kudo M., Harding J.J., Merle P., Rosmorduc O. (2022). Nivolumab versus sorafenib in advanced hepatocellular carcinoma (CheckMate 459): A randomised, multicentre, open-label, phase 3 trial. Lancet Oncol..

[31] He C., Peng W., Liu X., Li C., Li X., Wen T.-F. (2019). Post-treatment alpha-fetoprotein response predicts prognosis of patients with hepatocellular carcinoma: A meta-analysis. Medicine.

[32] Lee M.S., Ryoo B.Y., Hsu C.H., Numata K., Stein S., Verret W., Hack S.P., Spahn J., Liu B., Abdullah H. (2020). Atezolizumab with or without bevacizumab in unresectable hepatocellular carcinoma (GO30140): An open-label, multicentre, phase 1b study. Lancet Oncol..

[33] Zhu A.X., Dayyani F., Yen C.J., Ren Z., Bai Y., Meng Z., Pan H., Dillon P., Mhatre S.K., Gaillard V.E. (2022). Alpha-Fetoprotein as a Potential Surrogate Biomarker for Atezolizumab + Bevacizumab Treatment of Hepatocellular Carcinoma. Clin. Cancer Res..

[34] Tazzyman S., Lewis C.E., Murdoch C. (2009). Neutrophils: Key mediators of tumour angiogenesis. Int. J. Exp. Pathol..

[35] Zhou L., Zhang Z., Tian Y., Li Z., Liu Z., Zhu S. (2023). The critical role of platelet in cancer progression and metastasis. Eur. J. Med. Res..

[36] Jost-Brinkmann F., Demir M., Wree A., Luedde T., Loosen S.H., Müller T., Tacke F., Roderburg C., Mohr R. (2023). Atezolizumab plus bevacizumab in unresectable hepatocellular carcinoma: Results from a German real-world cohort. Aliment. Pharmacol. Ther..

[37] Wu Y.L., Fulgenzi C.A.M., D’Alessio A., Cheon J., Nishida N., Saeed A., Wietharn B., Cammarota A., Pressiani T., Personeni N. (2022). Neutrophil-to-Lymphocyte and Platelet-to-Lymphocyte Ratios as Prognostic Biomarkers in Unresectable Hepatocellular Carcinoma Treated with Atezolizumab plus Bevacizumab. Cancers.

[38] Dharmapuri S., Özbek U., Lin J.-Y., Sung M., Schwartz M., Branch A.D., Ang C. (2020). Predictive value of neutrophil to lymphocyte ratio and platelet to lymphocyte ratio in advanced hepatocellular carcinoma patients treated with anti–PD-1 therapy. Cancer Med..

[39] Zhang W., Tan Y., Li Y., Liu J. (2023). Neutrophil to Lymphocyte ratio as a predictor for immune-related adverse events in cancer patients treated with immune checkpoint inhibitors: A systematic review and meta-analysis. Front. Immunol..

[40] Pocino K., Stefanile A., Basile V., Napodano C., D’Ambrosio F., Di Santo R., Callà C.A.M., Gulli F., Saporito R., Ciasca G. (2022). Cytokines and Hepatocellular Carcinoma: Biomarkers of a Deadly Embrace. J. Pers. Med..

[41] Rico Montanari N., Anugwom C.M., Boonstra A., Debes J.D. (2021). The Role of Cytokines in the Different Stages of Hepatocellular Carcinoma. Cancers.

[42] Budhu A., Wang X.W. (2006). The role of cytokines in hepatocellular carcinoma. J. Leukoc. Biol..

[43] Liu H., Shen J., Lu K. (2017). IL-6 and PD-L1 blockade combination inhibits hepatocellular carcinoma cancer development in mouse model. Biochem. Biophys. Res. Commun..

[44] Huseni M.A., Wang L., Klementowicz J.E., Yuen K., Breart B., Orr C., Liu L.F., Li Y., Gupta V., Li C. (2023). CD8(+) T cell-intrinsic IL-6 signaling promotes resistance to anti-PD-L1 immunotherapy. Cell Rep. Med..

[45] Li J., Xu J., Yan X., Jin K., Li W., Zhang R. (2018). Targeting Interleukin-6 (IL-6) Sensitizes Anti-PD-L1 Treatment in a Colorectal Cancer Preclinical Model. Med. Sci. Monit..

[46] Yang H., Kang B., Ha Y., Lee S.H., Kim I., Kim H., Lee W.S., Kim G., Jung S., Rha S.Y. (2023). High serum IL-6 correlates with reduced clinical benefit of atezolizumab and bevacizumab in unresectable hepatocellular carcinoma. JHEP Rep..

[47] Myojin Y., Kodama T., Sakamori R., Maesaka K., Matsumae T., Sawai Y., Imai Y., Ohkawa K., Miyazaki M., Tanaka S. (2022). Interleukin-6 Is a Circulating Prognostic Biomarker for Hepatocellular Carcinoma Patients Treated with Combined Immunotherapy. Cancers.

[48] Suzuki T., Matsuura K., Suzuki Y., Okumura F., Nagura Y., Sobue S., Matoya S., Miyaki T., Kimura Y., Kusakabe A. (2024). Serum interleukin-6 levels at the start of the second course of atezolizumab plus bevacizumab therapy predict therapeutic efficacy in patients with advanced hepatocellular carcinoma: A multicenter analysis. J. Gastroenterol. Hepatol..

[49] Alfaro C., Sanmamed M.F., Rodríguez-Ruiz M.E., Teijeira Á., Oñate C., González Á., Ponz M., Schalper K.A., Pérez-Gracia J.L., Melero I. (2017). Interleukin-8 in cancer pathogenesis, treatment and follow-up. Cancer Treat. Rev..

[50] Tobin R.P., Jordan K.R., Kapoor P., Spongberg E., Davis D., Vorwald V.M., Couts K.L., Gao D., Smith D.E., Borgers J.S.W. (2019). IL-6 and IL-8 Are Linked with Myeloid-Derived Suppressor Cell Accumulation and Correlate with Poor Clinical Outcomes in Melanoma Patients. Front. Oncol..

[51] Rizzo M., Varnier L., Pezzicoli G., Pirovano M., Cosmai L., Porta C. (2022). IL-8 and its role as a potential biomarker of resistance to anti-angiogenic agents and immune checkpoint inhibitors in metastatic renal cell carcinoma. Front. Oncol..

[52] Zhang J., Yin Y., Tang J., Zhang Y., Tian Y., Sun F. (2024). Changes in Serum Interleukin-8 Levels Predict Response to Immune Checkpoint Inhibitors Immunotherapy in Unresectable Hepatocellular Carcinoma Patients. J. Inflamm. Res..

[53] Zou D., Song A., Yong W. (2023). Prognostic role of IL-8 in cancer patients treated with immune checkpoint inhibitors: A system review and meta-analysis. Front. Oncol..

[54] Chen J., Gingold J.A., Su X. (2019). Immunomodulatory TGF-β Signaling in Hepatocellular Carcinoma. Trends Mol. Med..

[55] Yamazaki K., Masugi Y., Sakamoto M. (2011). Molecular pathogenesis of hepatocellular carcinoma: Altering transforming growth factor-β signaling in hepatocarcinogenesis. Dig. Dis..

[56] Meindl-Beinker N.M., Matsuzaki K., Dooley S. (2012). TGF-β signaling in onset and progression of hepatocellular carcinoma. Dig. Dis..

[57] Feun L.G., Li Y.Y., Wu C., Wangpaichitr M., Jones P.D., Richman S.P., Madrazo B., Kwon D., Garcia-Buitrago M., Martin P. (2019). Phase 2 study of pembrolizumab and circulating biomarkers to predict anticancer response in advanced, unresectable hepatocellular carcinoma. Cancer.

[58] Kim Y., Yang H., Lee W.S., Cheon J., Sang Y.B., Kang B., Chon H.J., Kim C. (2023). High levels of baseline serum IL-10 are associated with reduced clinical benefit from first-line immune checkpoint inhibitor therapy in advanced renal cell carcinoma. J. Cancer.

[59] Babačić H., Lehtiö J., Pico de Coaña Y., Pernemalm M., Eriksson H. (2020). In-depth plasma proteomics reveals increase in circulating PD-1 during anti-PD-1 immunotherapy in patients with metastatic cutaneous melanoma. J. Immunother. Cancer.

[60] Chehrazi-Raffle A., Meza L., Alcantara M., Dizman N., Bergerot P., Salgia N., Hsu J., Ruel N., Salgia S., Malhotra J. (2021). Circulating cytokines associated with clinical response to systemic therapy in metastatic renal cell carcinoma. J. Immunother. Cancer.

[61] Costantini A., Takam Kamga P., Julie C., Corjon A., Dumenil C., Dumoulin J., Ouaknine J., Giraud V., Chinet T., Rottman M. (2020). Plasma Biomarkers Screening by Multiplex ELISA Assay in Patients with Advanced Non-Small Cell Lung Cancer Treated with Immune Checkpoint Inhibitors. Cancers.

[62] Boutsikou E., Domvri K., Hardavella G., Tsiouda D., Zarogoulidis K., Kontakiotis T. (2018). Tumour necrosis factor, interferon-gamma and interleukins as predictive markers of antiprogrammed cell-death protein-1 treatment in advanced non-small cell lung cancer: A pragmatic approach in clinical practice. Ther. Adv. Med. Oncol..

[63] Yamazaki N., Kiyohara Y., Uhara H., Iizuka H., Uehara J., Otsuka F., Fujisawa Y., Takenouchi T., Isei T., Iwatsuki K. (2017). Cytokine biomarkers to predict antitumor responses to nivolumab suggested in a phase 2 study for advanced melanoma. Cancer Sci..

[64] Chen C., Wang Z., Ding Y., Qin Y. (2023). Tumor microenvironment-mediated immune evasion in hepatocellular carcinoma. Front. Immunol..

[65] Sas Z., Cendrowicz E., Weinhäuser I., Rygiel T.P. (2022). Tumor Microenvironment of Hepatocellular Carcinoma: Challenges and Opportunities for New Treatment Options. Int. J. Mol. Sci..

[66] Klein E.A., Richards D., Cohn A., Tummala M., Lapham R., Cosgrove D., Chung G., Clement J., Gao J., Hunkapiller N. (2021). Clinical validation of a targeted methylation-based multi-cancer early detection test using an independent validation set. Ann. Oncol..

[67] Tran N.H., Kisiel J., Roberts L.R. (2021). Using cell-free DNA for HCC surveillance and prognosis. JHEP Rep..

[68] Fan R., Chen L., Zhao S., Yang H., Li Z., Qian Y., Ma H., Liu X., Wang C., Liang X. (2023). Novel, high accuracy models for hepatocellular carcinoma prediction based on longitudinal data and cell-free DNA signatures. J. Hepatol..

[69] Park S., Lee E.J., Rim C.H., Seong J. (2018). Plasma Cell-Free DNA as a Predictive Marker after Radiotherapy for Hepatocellular Carcinoma. Yonsei Med. J..

[70] Chen L., Wu T., Fan R., Qian Y.-S., Liu J.-F., Bai J., Zheng B., Liu X.-L., Zheng D., Du L.-T. (2024). Cell-free DNA testing for early hepatocellular carcinoma surveillance. eBioMedicine.

[71] Fu Y., Yang Z., Hu Z., Yang Z., Pan Y., Chen J., Wang J., Hu D., Zhou Z., Xu L. (2022). Preoperative serum ctDNA predicts early hepatocellular carcinoma recurrence and response to systemic therapies. Hepatol. Int..

[72] Matsumae T., Kodama T., Myojin Y., Maesaka K., Sakamori R., Takuwa A., Oku K., Motooka D., Sawai Y., Oshita M. (2022). Circulating Cell-Free DNA Profiling Predicts the Therapeutic Outcome in Advanced Hepatocellular Carcinoma Patients Treated with Combination Immunotherapy. Cancers.

[73] Hsu C.-H., Lu S., Abbas A., Guan Y., Zhu A.X., Aleshin A., Lee K.-H., Lee M.S., Mahipal A., Ryoo B.-Y. (2020). Longitudinal and personalized detection of circulating tumor DNA (ctDNA) for monitoring efficacy of atezolizumab plus bevacizumab in patients with unresectable hepatocellular carcinoma (HCC). J. Clin. Oncol..

[74] Zhan Q., Liu B., Situ X., Luo Y., Fu T., Wang Y., Xie Z., Ren L., Zhu Y., He W. (2023). New insights into the correlations between circulating tumor cells and target organ metastasis. Signal Transduct. Target. Ther..

[75] Nasr M.M., Lynch C.C. (2023). How circulating tumor cluster biology contributes to the metastatic cascade: From invasion to dissemination and dormancy. Cancer Metastasis Rev..

[76] Vasseur A., Kiavue N., Bidard F.C., Pierga J.Y., Cabel L. (2021). Clinical utility of circulating tumor cells: An update. Mol. Oncol..

[77] Pantel K., Speicher M.R. (2016). The biology of circulating tumor cells. Oncogene.

[78] Luo Q., Wang C., Peng B., Pu X., Cai L., Liao H., Chen K., Zhang C., Cheng Y., Pan M. (2020). Circulating Tumor-Cell-Associated White Blood Cell Clusters in Peripheral Blood Indicate Poor Prognosis in Patients with Hepatocellular Carcinoma. Front. Oncol..

[79] Espejo-Cruz M.L., González-Rubio S., Zamora-Olaya J., Amado-Torres V., Alejandre R., Sánchez-Frías M., Ciria R., De la Mata M., Rodríguez-Perálvarez M., Ferrín G. (2021). Circulating Tumor Cells in Hepatocellular Carcinoma: A Comprehensive Review and Critical Appraisal. Int. J. Mol. Sci..

[80] Chen J., Cao S.W., Cai Z., Zheng L., Wang Q. (2017). Epithelial-mesenchymal transition phenotypes of circulating tumor cells correlate with the clinical stages and cancer metastasis in hepatocellular carcinoma patients. Cancer Biomark..

[81] Chen V.L., Xu D., Wicha M.S., Lok A.S., Parikh N.D. (2020). Utility of Liquid Biopsy Analysis in Detection of Hepatocellular Carcinoma, Determination of Prognosis, and Disease Monitoring: A Systematic Review. Clin. Gastroenterol. Hepatol..

[82] Winograd P., Hou S., Court C.M., Lee Y.T., Chen P.J., Zhu Y., Sadeghi S., Finn R.S., Teng P.C., Wang J.J. (2020). Hepatocellular Carcinoma-Circulating Tumor Cells Expressing PD-L1 Are Prognostic and Potentially Associated with Response to Checkpoint Inhibitors. Hepatol. Commun..

[83] Su K., Guo L., He K., Rao M., Zhang J., Yang X., Huang W., Gu T., Xu K., Liu Y. (2022). PD-L1 expression on circulating tumor cells can be a predictive biomarker to PD-1 inhibitors combined with radiotherapy and antiangiogenic therapy in advanced hepatocellular carcinoma. Front. Oncol..

[84] Chen J.L., Guo L., Wu Z.Y., He K., Li H., Yang C., Han Y.W. (2024). Prognostic value of circulating tumor cells combined with neutrophil-lymphocyte ratio in patients with hepatocellular carcinoma. World J. Gastrointest. Oncol..

[85] Nosaka T., Murata Y., Akazawa Y., Tanaka T., Takahashi K., Naito T., Matsuda H., Ohtani M., Imamura Y., Nakamoto Y. (2024). Programmed Death Ligand 1 Expression in Circulating Tumor Cells as a Predictor and Monitor of Response to Atezolizumab plus Bevacizumab Treatment in Patients with Hepatocellular Carcinoma. Cancers.

[86] Lin D., Shen L., Luo M., Zhang K., Li J., Yang Q., Zhu F., Zhou D., Zheng S., Chen Y. (2021). Circulating tumor cells: Biology and clinical significance. Signal Transduct. Target Ther..

[87] Edd J.F., Mishra A., Smith K.C., Kapur R., Maheswaran S., Haber D.A., Toner M. (2022). Isolation of circulating tumor cells. iScience.

[88] Ahn J.C., Teng P.C., Chen P.J., Posadas E., Tseng H.R., Lu S.C., Yang J.D. (2021). Detection of Circulating Tumor Cells and Their Implications as a Biomarker for Diagnosis, Prognostication, and Therapeutic Monitoring in Hepatocellular Carcinoma. Hepatology.

[89] Li X., Li Y., Shao W., Li Z., Zhao R., Ye Z. (2020). Strategies for enrichment of circulating tumor cells. Transl. Cancer Res..

[90] Schöniger S., Jasani B. (2022). The PD-1/PD-L1 Pathway: A Perspective on Comparative Immuno-Oncology. Animals.

[91] Ai L., Xu A., Xu J. (2020). Roles of PD-1/PD-L1 Pathway: Signaling, Cancer, and Beyond. Adv. Exp. Med. Biol..

[92] Dermani F.K., Samadi P., Rahmani G., Kohlan A.K., Najafi R. (2019). PD-1/PD-L1 immune checkpoint: Potential target for cancer therapy. J. Cell Physiol..

[93] Nimmagadda S. (2020). Quantifying PD-L1 Expression to Monitor Immune Checkpoint Therapy: Opportunities and Challenges. Cancers.

[94] Doroshow D.B., Bhalla S., Beasley M.B., Sholl L.M., Kerr K.M., Gnjatic S., Wistuba I.I., Rimm D.L., Tsao M.S., Hirsch F.R. (2021). PD-L1 as a biomarker of response to immune-checkpoint inhibitors. Nat. Rev. Clin. Oncol..

[95] Zhu A.X., Finn R.S., Edeline J., Cattan S., Ogasawara S., Palmer D., Verslype C., Zagonel V., Fartoux L., Vogel A. (2018). Pembrolizumab in patients with advanced hepatocellular carcinoma previously treated with sorafenib (KEYNOTE-224): A non-randomised, open-label phase 2 trial. Lancet Oncol..

[96] El-Khoueiry A.B., Sangro B., Yau T., Crocenzi T.S., Kudo M., Hsu C., Kim T.Y., Choo S.P., Trojan J., Welling T.H.R. (2017). Nivolumab in patients with advanced hepatocellular carcinoma (CheckMate 040): An open-label, non-comparative, phase 1/2 dose escalation and expansion trial. Lancet.

[97] Yang Y., Chen D., Zhao B., Ren L., Huang R., Feng B., Chen H. (2023). The predictive value of PD-L1 expression in patients with advanced hepatocellular carcinoma treated with PD-1/PD-L1 inhibitors: A systematic review and meta-analysis. Cancer Med..

[98] Zhou X., Cao J., Topatana W., Xie T., Chen T., Hu J., Li S., Juengpanic S., Lu Z., Zhang B. (2023). Evaluation of PD-L1 as a biomarker for immunotherapy for hepatocellular carcinoma: Systematic review and meta-analysis. Immunotherapy.

[99] Cooper W.A., Russell P.A., Cherian M., Duhig E.E., Godbolt D., Jessup P.J., Khoo C., Leslie C., Mahar A., Moffat D.F. (2017). Intra- and Interobserver Reproducibility Assessment of PD-L1 Biomarker in Non-Small Cell Lung Cancer. Clin. Cancer Res..

[100] Ilie M., Long-Mira E., Bence C., Butori C., Lassalle S., Bouhlel L., Fazzalari L., Zahaf K., Lalvée S., Washetine K. (2016). Comparative study of the PD-L1 status between surgically resected specimens and matched biopsies of NSCLC patients reveal major discordances: A potential issue for anti-PD-L1 therapeutic strategies. Ann. Oncol..

[101] Kim H., Kwon H.J., Park S.Y., Park E., Chung J.H. (2017). PD-L1 immunohistochemical assays for assessment of therapeutic strategies involving immune checkpoint inhibitors in non-small cell lung cancer: A comparative study. Oncotarget.

[102] Wang M., Wang S., Trapani J.A., Neeson P.J. (2020). Challenges of PD-L1 testing in non-small cell lung cancer and beyond. J. Thorac. Dis..

[103] Lu L.C., Lee Y.H., Chang C.J., Shun C.T., Fang C.Y., Shao Y.Y., Liu T.H., Cheng A.L., Hsu C.H. (2019). Increased Expression of Programmed Death-Ligand 1 in Infiltrating Immune Cells in Hepatocellular Carcinoma Tissues after Sorafenib Treatment. Liver Cancer.

[104] Dong Z.Y., Wu S.P., Liao R.Q., Huang S.M., Wu Y.L. (2016). Potential biomarker for checkpoint blockade immunotherapy and treatment strategy. Tumour Biol..

[105] Ang C., Klempner S.J., Ali S.M., Madison R., Ross J.S., Severson E.A., Fabrizio D., Goodman A., Kurzrock R., Suh J. (2019). Prevalence of established and emerging biomarkers of immune checkpoint inhibitor response in advanced hepatocellular carcinoma. Oncotarget.

[106] Chan T.A., Yarchoan M., Jaffee E., Swanton C., Quezada S.A., Stenzinger A., Peters S. (2019). Development of tumor mutation burden as an immunotherapy biomarker: Utility for the oncology clinic. Ann. Oncol..

[107] Ning B., Liu Y., Wang M., Li Y., Xu T., Wei Y. (2022). The Predictive Value of Tumor Mutation Burden on Clinical Efficacy of Immune Checkpoint Inhibitors in Melanoma: A Systematic Review and Meta-Analysis. Front. Pharmacol..

[108] Rizvi N.A., Hellmann M.D., Snyder A., Kvistborg P., Makarov V., Havel J.J., Lee W., Yuan J., Wong P., Ho T.S. (2015). Cancer immunology. Mutational landscape determines sensitivity to PD-1 blockade in non-small cell lung cancer. Science.

[109] Rosenberg J.E., Hoffman-Censits J., Powles T., van der Heijden M.S., Balar A.V., Necchi A., Dawson N., O’Donnell P.H., Balmanoukian A., Loriot Y. (2016). Atezolizumab in patients with locally advanced and metastatic urothelial carcinoma who have progressed following treatment with platinum-based chemotherapy: A single-arm, multicentre, phase 2 trial. Lancet.

[110] Ricciuti B., Wang X., Alessi J.V., Rizvi H., Mahadevan N.R., Li Y.Y., Polio A., Lindsay J., Umeton R., Sinha R. (2022). Association of High Tumor Mutation Burden in Non–Small Cell Lung Cancers with Increased Immune Infiltration and Improved Clinical Outcomes of PD-L1 Blockade Across PD-L1 Expression Levels. JAMA Oncol..

[111] Xu J., Zhang Y., Jia R., Yue C., Chang L., Liu R., Zhang G., Zhao C., Zhang Y., Chen C. (2019). Anti-PD-1 Antibody SHR-1210 Combined with Apatinib for Advanced Hepatocellular Carcinoma, Gastric, or Esophagogastric Junction Cancer: An Open-label, Dose Escalation and Expansion Study. Clin. Cancer Res..

[112] Zhu A.X., Abbas A.R., de Galarreta M.R., Guan Y., Lu S., Koeppen H., Zhang W., Hsu C.-H., He A.R., Ryoo B.-Y. (2022). Molecular correlates of clinical response and resistance to atezolizumab in combination with bevacizumab in advanced hepatocellular carcinoma. Nat. Med..

[113] Spahn S., Roessler D., Pompilia R., Gabernet G., Gladstone B.P., Horger M., Biskup S., Feldhahn M., Nahnsen S., Hilke F.J. (2020). Clinical and Genetic Tumor Characteristics of Responding and Non-Responding Patients to PD-1 Inhibition in Hepatocellular Carcinoma. Cancers.

[114] Kavun A., Veselovsky E., Lebedeva A., Belova E., Kuznetsova O., Yakushina V., Grigoreva T., Mileyko V., Fedyanin M., Ivanov M. (2023). Microsatellite Instability: A Review of Molecular Epidemiology and Implications for Immune Checkpoint Inhibitor Therapy. Cancers.

[115] Nguyen L.H., Goel A., Chung D.C. (2020). Pathways of Colorectal Carcinogenesis. Gastroenterology.

[116] Mandal R., Samstein R.M., Lee K.W., Havel J.J., Wang H., Krishna C., Sabio E.Y., Makarov V., Kuo F., Blecua P. (2019). Genetic diversity of tumors with mismatch repair deficiency influences anti-PD-1 immunotherapy response. Science.

[117] Wang Z., Zhao J., Wang G., Zhang F., Zhang Z., Zhang F., Zhang Y., Dong H., Zhao X., Duan J. (2018). Comutations in DNA Damage Response Pathways Serve as Potential Biomarkers for Immune Checkpoint Blockade. Cancer Res..

[118] Yan L., Zhang W. (2018). Precision medicine becomes reality—Tumor type-agnostic therapy. Cancer Commun..

[119] Chang L., Chang M., Chang H.M., Chang F. (2018). Microsatellite Instability: A Predictive Biomarker for Cancer Immunotherapy. Appl. Immunohistochem. Mol. Morphol..

[120] Marcus L., Lemery S.J., Keegan P., Pazdur R. (2019). FDA Approval Summary: Pembrolizumab for the Treatment of Microsatellite Instability-High Solid Tumors. Clin. Cancer Res..

[121] Food U., Administration D. (2017). FDA Approves First Cancer Treatment for Any Solid Tumor with a Specific Genetic Feature.

[122] Ando Y., Yamauchi M., Suehiro Y., Yamaoka K., Kosaka Y., Fuji Y., Uchikawa S., Kodama K., Morio K., Fujino H. (2020). Complete response to pembrolizumab in advanced hepatocellular carcinoma with microsatellite instability. Clin. J. Gastroenterol..

[123] Kawaoka T., Ando Y., Yamauchi M., Suehiro Y., Yamaoka K., Kosaka Y., Fuji Y., Uchikawa S., Morio K., Fujino H. (2020). Incidence of microsatellite instability-high hepatocellular carcinoma among Japanese patients and response to pembrolizumab. Hepatol. Res..

[124] Eso Y., Shimizu T., Takeda H., Takai A., Marusawa H. (2020). Microsatellite instability and immune checkpoint inhibitors: Toward precision medicine against gastrointestinal and hepatobiliary cancers. J. Gastroenterol..

[125] Zheng X., Jin W., Wang S., Ding H. (2021). Progression on the Roles and Mechanisms of Tumor-Infiltrating T Lymphocytes in Patients with Hepatocellular Carcinoma. Front. Immunol..

[126] Sangro B., Melero I., Wadhawan S., Finn R.S., Abou-Alfa G.K., Cheng A.L., Yau T., Furuse J., Park J.W., Boyd Z. (2020). Association of inflammatory biomarkers with clinical outcomes in nivolumab-treated patients with advanced hepatocellular carcinoma. J. Hepatol..

[127] Zhang W., Gong C., Peng X., Bi X., Sun Y., Zhou J., Wu F., Zeng H., Wang Y., Zhou H. (2022). Serum Concentration of CD137 and Tumor Infiltration by M1 Macrophages Predict the Response to Sintilimab plus Bevacizumab Biosimilar in Advanced Hepatocellular Carcinoma Patients. Clin. Cancer Res..

[128] Kuwano A., Yada M., Miyazaki Y., Tanaka K., Kurosaka K., Ohishi Y., Masumoto A., Motomura K. (2023). Tumor-infiltrating CD8(+) T cells as a biomarker for chemotherapy efficacy in unresectable hepatocellular carcinoma. Oncol. Lett..

[129] Duffy A.G., Ulahannan S.V., Makorova-Rusher O., Rahma O., Wedemeyer H., Pratt D., Davis J.L., Hughes M.S., Heller T., ElGindi M. (2017). Tremelimumab in combination with ablation in patients with advanced hepatocellular carcinoma. J. Hepatol..

[130] Ng H.H.M., Lee R.Y., Goh S., Tay I.S.Y., Lim X., Lee B., Chew V., Li H., Tan B., Lim S. (2020). Immunohistochemical scoring of CD38 in the tumor microenvironment predicts responsiveness to anti-PD-1/PD-L1 immunotherapy in hepatocellular carcinoma. J. Immunother. Cancer.

[131] Cheung C.C.L., Seah Y.H.J., Fang J., Orpilla N.H.C., Lau M.C., Lim C.J., Lim X., Lee J., Lim J.C.T., Lim S. (2023). Immunohistochemical scoring of LAG-3 in conjunction with CD8 in the tumor microenvironment predicts response to immunotherapy in hepatocellular carcinoma. Front. Immunol..

[132] Zheng C., Zheng L., Yoo J.-K., Guo H., Zhang Y., Guo X., Kang B., Hu R., Huang J.Y., Zhang Q. (2017). Landscape of Infiltrating T Cells in Liver Cancer Revealed by Single-Cell Sequencing. Cell.

[133] Ren X., Zhang Z. (2019). Understanding tumor-infiltrating lymphocytes by single cell RNA sequencing. Adv. Immunol..

[134] Peeters F., Cappuyns S., Piqué-Gili M., Phillips G., Verslype C., Lambrechts D., Dekervel J. (2024). Applications of single-cell multi-omics in liver cancer. JHEP Rep..

[135] Chan L.-K., Tsui Y.-M., Ho D.W.-H., Ng I.O.-L. (2022). Cellular heterogeneity and plasticity in liver cancer. Semin. Cancer Biol..

[136] Kalasekar S.M., VanSant-Webb C.H., Evason K.J. (2021). Intratumor Heterogeneity in Hepatocellular Carcinoma: Challenges and Opportunities. Cancers.

[137] Kurebayashi Y., Ojima H., Tsujikawa H., Kubota N., Maehara J., Abe Y., Kitago M., Shinoda M., Kitagawa Y., Sakamoto M. (2018). Landscape of immune microenvironment in hepatocellular carcinoma and its additional impact on histological and molecular classification. Hepatology.

[138] Losic B., Craig A.J., Villacorta-Martin C., Martins-Filho S.N., Akers N., Chen X., Ahsen M.E., von Felden J., Labgaa I., DʹAvola D. (2020). Intratumoral heterogeneity and clonal evolution in liver cancer. Nat. Commun..

[139] Kim M.S., Xu A., Haslam A., Prasad V. (2022). Quality of biomarker defined subgroups in FDA approvals of PD-1/PD-L1 inhibitors 2014 to 2020. Int. J. Cancer.

[140] Ballman K.V. (2015). Biomarker: Predictive or Prognostic?. J. Clin. Oncol..

[141] Oldenhuis C.N., Oosting S.F., Gietema J.A., de Vries E.G. (2008). Prognostic versus predictive value of biomarkers in oncology. Eur. J. Cancer.

[142] Song B.G., Goh M.J., Kang W., Sinn D.H., Gwak G.Y., Choi M.S., Lee J.H., Paik Y.H. (2024). Analysis of Factors Predicting the Real-World Efficacy of Atezolizumab and Bevacizumab in Patients with Advanced Hepatocellular Carcinoma. Gut Liver.

[143] Kunichika H., Minamiguchi K., Tachiiri T., Shimizu K., Taiji R., Yamada A., Nakano R., Irizato M., Yamauchi S., Marugami A. (2024). Prediction of Efficacy for Atezolizumab/Bevacizumab in Unresectable Hepatocellular Carcinoma with Hepatobiliary-Phase Gadolinium Ethoxybenzyl-Diethylenetriaminepentaacetic Acid MRI. Cancers.

[144] Haber P.K., Castet F., Torres-Martin M., Andreu-Oller C., Puigvehí M., Miho M., Radu P., Dufour J.-F., Verslype C., Zimpel C. (2023). Molecular Markers of Response to Anti-PD1 Therapy in Advanced Hepatocellular Carcinoma. Gastroenterology.

[145] Liu Z., Yang L., Liu C., Wang Z., Xu W., Lu J., Wang C., Xu X. (2024). Identification and validation of immune-related gene signature models for predicting prognosis and immunotherapy response in hepatocellular carcinoma. Front. Immunol..

[146] Jin X., Zhou K., Zhang R., Li J., Guo M., Qiao H., Wu M., Cao X., Dong G., Zhang S. (2024). Construction and validation of prognostic signature for transcription factors regulating T cell exhaustion in hepatocellular carcinoma. Medicine.

